# How does self-efficacy promote physical activity among adolescents? The mediating roles of task and ego orientations

**DOI:** 10.3389/fpsyg.2026.1929519

**Published:** 2026-10-01

**Authors:** Pengpeng Xu, Li Ma, Ke Jia

**Affiliations:** 1 Sport Art College, Guangzhou Sport University, Guangzhou, China; 2 School of Sport Media, Guangzhou Sport University, Guangzhou, China

**Keywords:** adolescent, ego orientation, physical activity, self-efficacy, self-efficacy theory, task orientation

## Abstract

**Introduction:**

Regular participation in physical activity is essential for adolescents’ overall development, yet physical inactivity remains a prevalent public health concern. To elucidate the motivational mechanisms underlying physical activity among Chinese adolescents, this study investigated the parallel mediating roles of task and ego orientations in the relationship between self-efficacy and physical activity, alongside potential gender differences.

**Methods:**

A cross-sectional survey was conducted among 1,194 junior high school students (576 boys, 618 girls; *M* age = 13.27 years) in China. Data on physical activity self-efficacy, achievement goal orientations, and physical activity were analyzed using structural equation modeling (SEM) and multi-group analyses.

**Results:**

Self-efficacy directly and positively predicted physical activity. Although the direct path from task orientation to physical activity was not significant, both task and ego orientations functioned as parallel mediators with significant indirect effects. Furthermore, multi-group SEM established structural invariance across genders and revealed a significant moderating role of gender that the predictive path from self-efficacy to ego orientation was significantly stronger in boys than in girls. In conclusion, self-efficacy promotes adolescents’ physical activity through both direct and dual-motivational indirect pathways.

**Discussion:**

The findings suggest that the behavioral translation of task orientation is constrained by academic pressures, whereas ego orientation acts as a robust catalyst driven by peer competition and gender socialization. Interventions should employ gender-sensitive strategies to optimize adolescents’ self-efficacy and achievement motives.

## Introduction

Regular participation in physical activity is essential for adolescents’ physical (Kim et al., 2021; Lewis et al., 2016), psychological, and cognitive development (Chen et al., 2023). Sufficient physical activity contributes to cardiovascular health, healthy weight maintenance, bone development, and psychological wellbeing, while also supporting cognitive functioning and academic performance (Chen et al., 2020a, 2020b). Despite these well documented benefits, physical inactivity remains a major public health concern worldwide. According to recent estimates from the World Health Organization, more than four fifths of adolescents aged 11–17 years fail to achieve the recommended 60 min of moderate to vigorous physical activity per day (Ahmed et al., 2022). This trend is particularly concerning because physical inactivity during adolescence often persists into adulthood and increases the risk of numerous adverse health outcomes (Li et al., 2019).

In China, the lifestyle of adolescents is increasingly characterized by prolonged sedentary behaviors, extensive homework assignments, after-school tutoring, and screen-based leisure activities, all of which have substantially reduced the time available for physical activity (Meng et al., 2025). These academic demands, intensified by the pressure of the high-stakes senior high school entrance examination (Zhongkao), often crowd out discretionary time that could otherwise be allocated to sports and active recreation (Li et al., 2026). Although the government has launched national health strategies such as the Healthy China 2030 initiative to prioritize school-based physical activity, empirical evidence suggests that these macro-level efforts have not translated into commensurate behavioral changes (Chen et al., 2019a, 2019b). Despite improvements in school facilities and policy mandates, the magnitude of behavioral change remained modest, with effect sizes significantly smaller than those achieved by psychologically informed interventions (Ma et al., 2020). According to previous research, only about half of school-aged youth meet the daily recommendation of 60 min of moderate to vigorous physical activity (Chen et al., 2023). These statistics reveal a critical policy-behavior gap, suggesting that environmental and structural interventions alone are insufficient to drive sustained behavioral change. To bridge this gap, it is imperative to understand the micro-level psychological mechanisms—particularly the intrinsic motivational and cognitive factors—that translate policy intentions into actual behavioral engagement among adolescents.

Physical activity behavior is influenced by multiple factors operating at individual, interpersonal, environmental, and societal levels (Lu et al., 2017). Among these determinants, individual psychological factors are considered particularly important because they are closely related to behavioral initiation and maintenance (Spence et al., 2010; Williams and French, 2011). Self-efficacy, defined as an individual’s belief in their capability to successfully perform a specific behavior (Bandura, 1977), has consistently emerged as one of the strongest psychological predictors of adolescent physical activity (Hamilton et al., 2016). According to Self-Efficacy Theory, individuals who possess stronger efficacy beliefs are more likely to initiate challenging behaviors, persist in the face of obstacles, and maintain effort over time (Dishman et al., 2005). Empirical evidence has repeatedly demonstrated positive associations between self-efficacy and physical activity participation among adolescents (Kim et al., 2021; Yiming et al., 2023). However, although the predictive role of self-efficacy is well established, less is known about the motivational processes through which self-efficacy influences physical activity behavior. One promising theoretical lens for unpacking these processes is Achievement Goal Theory, which posits that individuals define competence and success according to different evaluative criteria.

One potential explanation may be found in achievement goal orientations. Achievement Goal Theory proposes that individuals adopt different criteria for defining competence and success in achievement settings (Pérez-Quero et al., 2023). Within sport and physical activity contexts, achievement goal orientations are commonly conceptualized as task orientation and ego orientation (Cumming and Hall, 2004). Task oriented individuals define success through personal improvement, effort, and skill mastery, whereas ego oriented individuals evaluate success through social comparison and superior performance relative to others (Sánchez et al., 2024). Previous research has shown that both forms of achievement orientation are associated with motivation, persistence, and behavioral engagement in sport and physical activity settings. Importantly, achievement goal orientations may also be shaped by efficacy beliefs. Adolescents who perceive themselves as capable of performing successfully may be more likely to pursue achievement goals and actively engage in physical activity. Importantly, achievement goal orientations may also be shaped by efficacy beliefs. According to Bandura’s social cognitive perspective, efficacy beliefs shape the types of anticipatory scenarios individuals construct and the goals they set (Gao et al., 2010). Specifically, adolescents with high self-efficacy are less constrained by the fear of failure, which enables them to focus on effort and skill mastery (task orientation). Simultaneously, a strong belief in their capabilities may also provide the confidence needed to engage in social comparison and strive for normative competence (ego orientation; Cumming and Hall, 2004; Rao, 2008). Therefore, achievement goal orientations may represent important motivational mechanisms linking self-efficacy to physical activity.

Although previous studies have separately examined the relationships among self-efficacy, achievement goal orientations, and physical activity, several important gaps remain (Cumming and Hall, 2004; Pérez-Quero et al., 2023). First, relatively few studies have simultaneously examined task orientation and ego orientation as parallel mediators in the association between self-efficacy and physical activity. As a result, it remains unclear whether self-efficacy promotes physical activity through mastery related motivational processes, performance related motivational processes, or both. Second, most existing studies have focused on direct associations between psychological variables and physical activity (Biddle et al., 2011), providing limited understanding of the underlying motivational pathways. Third, evidence regarding gender differences in these relationships remains inconclusive. Given that boys and girls may differ in their efficacy beliefs, achievement goals, and physical activity participation patterns, examining the invariance of the proposed model across gender groups may provide additional insight into the generalizability of the underlying mechanisms.

Therefore, the present study investigated the relationship between self-efficacy and physical activity among Chinese adolescents and examined the mediating roles of task orientation and ego orientation (as shown in Figure 1). Drawing upon Self-Efficacy Theory, a parallel mediation model was proposed in which self-efficacy influences physical activity directly and indirectly through achievement goal orientations. In addition, gender differences in the proposed pathways were examined through multi group analyses. Based on the theoretical framework and empirical evidence reviewed above, the following hypotheses were formulated to correspond directly to the structural paths specified in Figure 1:

**Figure 1:**
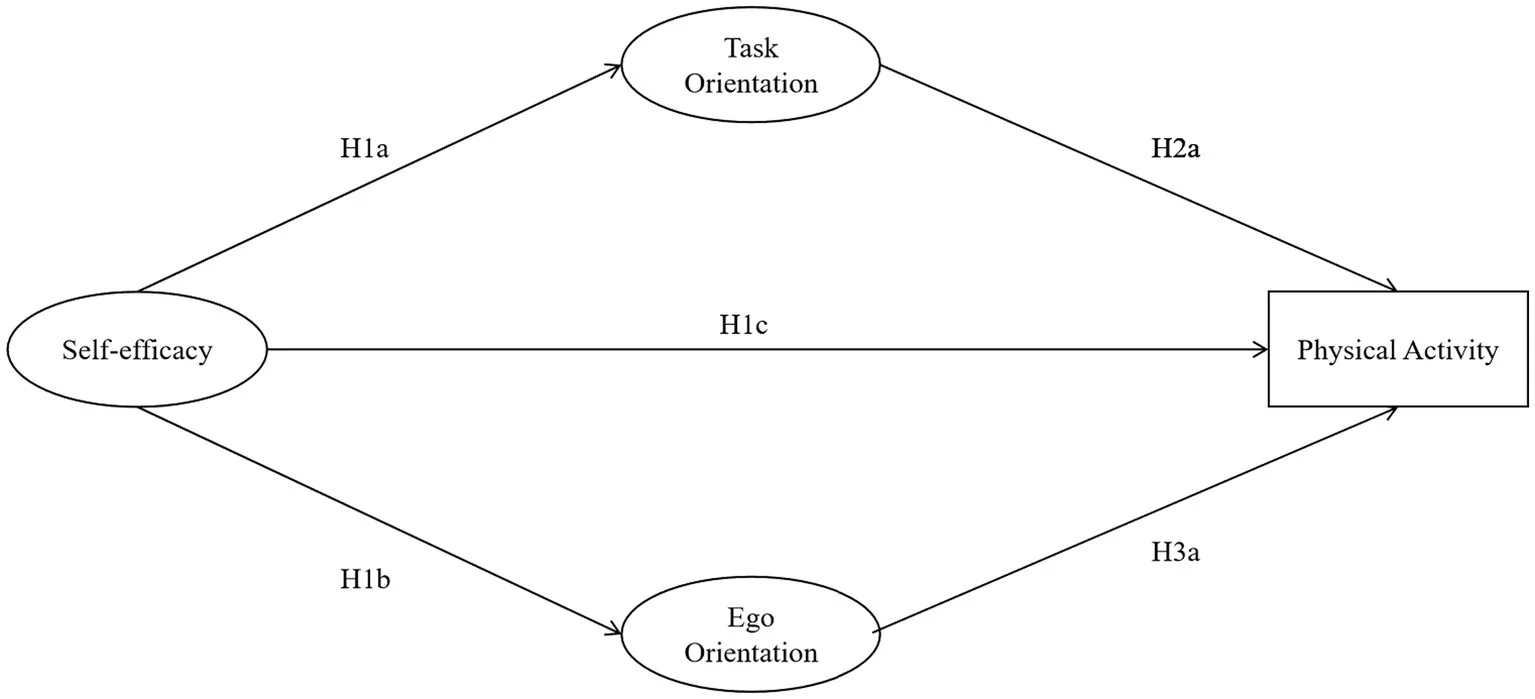
Hypothesized parallel mediation model.

*H1a*: Self-efficacy will be positively associated with task orientation.

*H1b*: Self-efficacy will be positively associated with ego orientation.

*H1c*: Self-efficacy will exert a significant and positive direct effect on physical activity.

*H2a*: Task orientation will be positively associated with physical activity.

*H3a*: Ego orientation will be positively associated with physical activity.

## Methods

### Participants

A convenience sampling strategy was used to recruit junior high school students from five cities in southern China: Guangzhou, Foshan, Huizhou, and Shantou in Guangdong Province, and Nanping in Fujian Province. These cities were selected to represent a diverse spectrum of socioeconomic contexts, with 2025 regional GDPs ranging from approximately 21.90 billion RMB (Nanping) to 320.39 billion RMB (Guangzhou), encompassing different levels of urbanization, educational resources, and physical activity infrastructure. This deliberate variation was intended to enhance the heterogeneity of the sample, thereby improving the generalizability of the findings beyond any single urban setting.

A total of 1,443 questionnaires were initially collected. However, to mitigate the risk of acquiescence bias, where respondents might select affirmative responses as a shortcut, potentially not reflecting their true opinions, portions of the questionnaire exhibiting consistent affirmative answers and any blank questionnaires were omitted from the analysis. After excluding questionnaires with incomplete responses or evident patterned answering, 1,194 valid questionnaires were retained for analysis, yielding an effective response rate of 82.7%. The final sample included 576 boys and 618 girls, with a mean age of 13.27 years (*SD* = 1.00).

### Measures

#### Self-efficacy

Physical activity self-efficacy was assessed using the Chinese version of the Self-Perception of Adequacy in Physical Activity Scale for Adolescents (Motl et al., 2002) and later adapted for Chinese adolescents (Chen et al., 2019a, 2019b). The scale consists of 8 items rated on a 5-point Likert scale ranging from 1 (strongly disagree) to 5 (strongly agree). Higher scores indicate stronger self-efficacy for engaging in physical activity. Previous studies have demonstrated satisfactory reliability and validity of the Chinese version across adolescent populations with different genders and educational backgrounds. In the present study, Cronbach’s α was 0.885, indicating good internal consistency.

#### Achievement goal orientations

Task orientation and ego orientation were measured using the Task and Ego Orientation in Sport Questionnaire (Ma and Monsma, 2016). The questionnaire is widely used to assess individuals’ perceptions of success in sport settings and contains two dimensions: task orientation and ego orientation. Items are rated on a 5-point Likert scale, with higher scores indicating a stronger tendency toward the corresponding goal orientation. The Chinese version has shown good psychometric properties among adolescent samples. In the present study, Cronbach’s α was 0.862 for task orientation, and 0.840 for ego orientation, respectively, demonstrating good reliability.

#### Physical activity

Physical activity was assessed using the short form of the International Physical Activity Questionnaire (Craig et al., 2003; Lee et al., 2011). The Chinese version of the IPAQ-SF has demonstrated acceptable reliability and validity in Chinese populations (Liu et al., 2025). The questionnaire includes 7 items, with six items assessing physical activity behavior and one item assessing sedentary behavior. Weekly metabolic equivalent minutes were calculated by multiplying the assigned MET value for each activity type by weekly frequency and daily duration. According to the IPAQ scoring protocol, walking was assigned a MET value of 3.3, moderate-intensity activity a value of 4.0, and vigorous-intensity activity a value of 8.0. To improve the robustness of the structural equation modeling analyses, total physical activity scores were standardized using z-score transformation before model testing.

#### Demographic variables

We collected basic demographic information from participants, including their self-reported gender (boy = 1, girl = 2) and age.

### Data analysis

Descriptive statistics and bivariate correlations were computed using SPSS 26.0. Harman single factor test was performed to assess the potential influence of common method bias. Prior to model testing, univariate normality was examined based on skewness and kurtosis values. The physical activity variable exhibited significant positive skewness (skewness = 1.166, *SE* = 0.070) and kurtosis (kurtosis = 1.050, *SE* = 0.140), indicating a substantial deviation from the normal distribution. This pattern is consistent with previous research using the IPAQ-SF, which has shown that physical activity data in adolescent populations are typically positively skewed. Given this violation of the normality assumption, we employed the robust maximum likelihood (MLM) estimator in Mplus to obtain standard errors and model fit statistics that are robust to non-normality. In addition, indirect effects were examined using bias-corrected bootstrap confidence intervals based on 5,000 resamples, as this approach does not assume multivariate normality and provides more accurate confidence intervals for mediated effects.

Model fit was evaluated using the chi square statistic, the Comparative Fit Index, the Tucker Lewis Index, the Root Mean Square Error of Approximation, and the Standardized Root Mean Square Residual. Following the recommendations of Hu and Bentler, values of CFI and TLI equal to or greater than 0.95, RMSEA equal to or less than 0.06, and SRMR equal to or less than 0.08 were considered indicative of good fit. Indirect effects were examined using bias corrected bootstrap confidence intervals based on 5,000 resamples.

### Procedure

This study was approved by the university Ethics Committee (Approval No. 2025LLCL-165). Data collection was conducted in December 2025 in China. After obtaining permission from school administrators, informed consent was obtained from parents or guardians as well as assent from the participating students. Participants completed paper-based questionnaires in their classrooms under the supervision of trained researchers. The survey took approximately 10 to 15 min to complete. Participants were informed that their responses would remain confidential and would be used only for research purposes. Upon completion of the questionnaire, each participant received a small token of appreciation, such as a bookmark or keychain.

## Results

### Descriptive statistics and correlations

Descriptive statistics, reliability coefficients, and bivariate correlations among the study variables are presented in Table 1. Self-efficacy was positively correlated with ego orientation (*r* = 0.348, *p* < 0.001), task orientation (*r* = 0.536, *p* < 0.001), and physical activity (*r* = 0.311, *p* < 0.001). Ego orientation was positively associated with task orientation (*r* = 0.613, *p* < 0.001) and physical activity (*r* = 0.225, *p* < 0.001). Task orientation was also positively correlated with physical activity (*r* = 0.279, *p* < 0.001). Age was negatively associated with ego orientation (*r* = −0.086, *p* < 0.01) and physical activity (*r* = −0.110, *p* < 0.001). Gender (coded as 1 = boy, 2 = girl) was negatively correlated with self-efficacy (*r* = −0.170, *p* < 0.001), task orientation (*r* = −0.079, *p* < 0.01), and physical activity (*r* = −0.084, *p* < 0.01), indicating that female adolescents reported lower scores on these variables compared to their male counterparts. Overall, the observed correlations were in the expected directions and provided preliminary support for the proposed model.

**Table 1:** Descriptive statistics and correlations of key variables.

No	Variables	*M*	*SD*	Ske	Kur	*α*	1	2	3	4	5
1	Self-efficacy	3.404	0.867	−0.032	0.150	0.885					
2	Ego orientation	3.355	0.881	−0.220	−0.254	0.840	0.348^***^				
3	Task orientation	3.657	0.758	−0.447	0.563	0.862	0.536^***^	0.613^***^			
4	Physical activity	−0.060	0.873	1.166	1.050	/	0.311^***^	0.225^***^	0.279^***^		
5	Age	13.267	0.999	/	/	/	0.016	−0.086^**^	−0.043	−0.110^***^	
6	Gender	/	/	/	/	/	−0.170^***^	−0.037	−0.079^**^	−0.084^**^	−0.026

^*^p < 0.05, ^**^p < 0.01, ^***^p < 0.001. Ske = Skewness, Kur = Kurtosis.

### Common method Bias and multicollinearity

Given that all variables were assessed using self-report measures, Harman’s single-factor test was conducted to examine the potential influence of common method bias (Fuller et al., 2016). The results indicated that the first unrotated factor did not account for the majority of the total variance, suggesting that common method bias was unlikely to be a serious concern in the present study.

Multicollinearity was examined using tolerance values and variance inflation factors (Romeo et al., 2017). As shown in Table 2, the tolerance values ranged from 0.507 to 0.713, while the variance inflation factors ranged from 1.403 to 1.973. All values were well within the recommended thresholds, indicating that multicollinearity was not a concern.

**Table 2:** Results of VIF.

No	Variables	Tolerance	VIF
1	Self-efficacy	0.713	1.403
2	Ego orientation	0.623	1.604
3	Task orientation	0.507	1.973

### Structural equation modeling results

The hypothesized structural model was tested using structural equation modeling, and the standardized path coefficients are presented in Figure 1. As shown in Table 3, self-efficacy was positively associated with task orientation (*β* = 0.630, *SE* = 0.025, *p* < 0.001), ego orientation (*β* = 0.440, *SE* = 0.031, *p* < 0.001), and physical activity (*β* = 0.260, SE = 0.052, *p* < 0.001; Figure 2).

**Table 3:** Results of structural equation modeling.

Paths	*β*	*SE*	*p*	95% CI Lower	95% CI Upper
*H1a*: Self-efficacy → Task orientation	0.630	0.025	<0.001	0.589	0.671
*H1b*: Self-efficacy → Ego orientation	0.440	0.031	<0.001	0.389	0.491
*H1c*: Self-efficacy → Physical activity	0.260	0.052	<0.001	0.175	0.345
*H2a*: Task orientation → Physical activity	0.084	0.044	0.056	0.012	0.156
*H3a*: Ego orientation → Physical activity	0.081	0.019	0.019	0.024	0.138
Total effects: Self-efficacy → Physical activity	0.349	0.028	<0.001	0.302	0.395
Total indirect effects: Self-efficacy → Physical activity	0.089	0.039	0.023	0.024	0.153
Self-efficacy → Task orientation → Physical activity	0.053	0.028	0.056	0.007	0.098
Self-efficacy → Ego orientation → Physical activity	0.036	0.015	0.021	0.010	0.061

**Figure 2:**
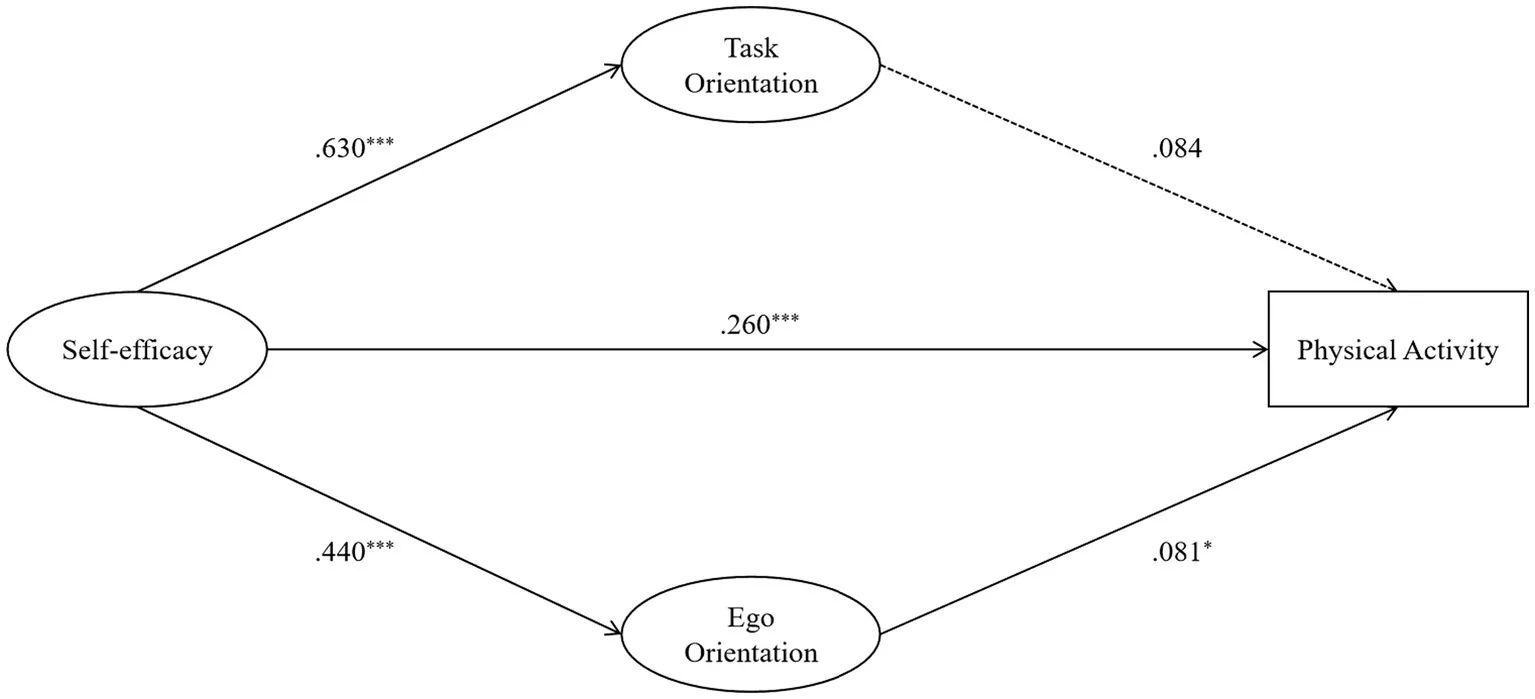
Standardized structural model for the total sample.

The direct path from task orientation to physical activity did not reach conventional statistical significance (*β* = 0.084, *p* = 0.056), whereas ego orientation significantly and positively predicted physical activity (*β* = 0.081, *p* = 0.019). The total effect of self-efficacy on physical activity was significant (*β* = 0.349, *p* < 0.001), as was the total indirect effect through both goal orientations (*β* = 0.089, *p* = 0.023, 95% CI [0.024, 0.153]). Regarding the specific indirect pathways, the bias corrected bootstrap analysis with 5,000 resamples revealed that the mediating role of task orientation was supported, as its specific indirect effect (*β* = 0.053, 95% CI [0.007, 0.098]) did not include zero. Similarly, the indirect pathway via ego orientation was also supported (*β* = 0.036, 95% CI [0.010, 0.061]).

### Measurement invariance and gender differences

Before conducting gender comparisons, measurement invariance across gender was examined for self-efficacy, task orientation, and ego orientation. The results supported configural, metric, and scalar invariance for all three constructs. Changes in CFI and RMSEA across increasingly constrained models remained within the recommended criteria, indicating that the measures operated equivalently across male and female participants (Table 4).

**Table 4:** Measurement invariance of gender.

Model	Invariance	χ^2^	*df*	RMSEA	CFI	SRMR	△RMSEA	△CFI
1	Configural invariance	143.412	40	0.065	0.967	0.035	/	/
	Metric invariance	156.235	47	0.062	0.965	0.038	−0.003	−0.002
	Scalar invariance	186.023	54	0.063	0.957	0.043	0.001	−0.008
2	Configural invariance	43.700	28	0.030	0.992	0.022	/	/
	Metric invariance	51.173	34	0.029	0.992	0.031	−0.001	<0.001
	Scalar invariance	68.210	40	0.034	0.986	0.035	0.005	−0.006
3	Configural invariance	113.222	18	0.093	0.952	0.038	/	/
	Metric invariance	130.439	23	0.088	0.946	0.046	−0.005	−0.006
	Scalar invariance	153.249	28	0.086	0.937	0.050	−0.002	−0.009

Model 1 demonstrates the measurement invariance of self-efficacy across gender groups. Model 2 demonstrates the measurement invariance of task orientation across gender groups. Model 3 demonstrates the measurement invariance of ego orientation across gender groups.

Subsequently, multi-group structural equation modeling was conducted to examine potential gender differences in structural relationships, and the group-specific path coefficients are presented in Figure 3. The path from self-efficacy to task orientation was significant for both boys (*β* = 0.655, *p* < 0.001) and girls (*β* = 0.478, *p* < 0.001), with no significant difference between groups (*β*_diff_ = 0.082, *p* = 0.209). Similarly, the direct association between self-efficacy and physical activity did not differ significantly between boys and girls (*β*_diff_ = 0.179, *p* = 0.124).

**Figure 3:**
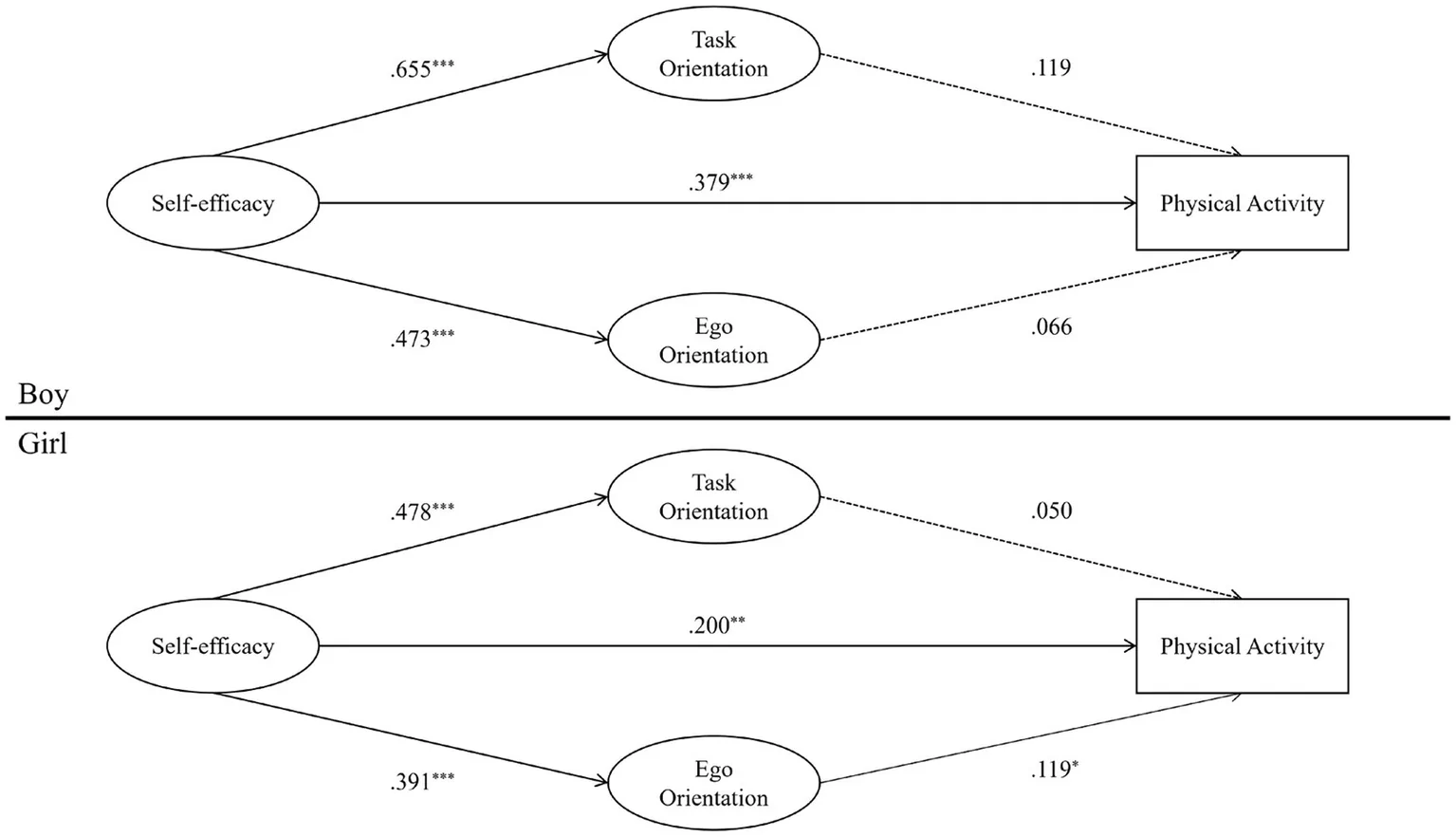
Multi-group structural model by gender.

A significant gender difference was observed in the association between self-efficacy and ego orientation (*β*_diff_ = 0.178, *p* = 0.003), indicating that this relationship was stronger among boys than girls. No significant gender differences were found for the associations between task orientation and physical activity (*β*_diff_ = −0.054, *p* = 0.485) or between ego orientation and physical activity (*β*_diff_ = 0.069, *p* = 0.520; Table 5).

**Table 5:** Multi group comparison of structural paths by gender.

Route	Boy			Girl			Comparison		
	*β*	*SE*	*p*	*β*	*SE*	*p*	*β*	*SE*	*p*
Self-efficacy → Task orientation	0.655	0.051	<0.001	0.478	0.044	<0.001	0.082	0.065	0.209
Self-efficacy → Ego orientation	0.473	0.052	<0.001	0.391	0.050	<0.001	0.178	0.061	0.003
Self-efficacy → Physical activity	0.379	0.093	<0.001	0.200	0.070	0.004	0.179	0.116	0.124
Task orientation → Physical activity	0.119	0.080	0.139	0.050	0.070	0.476	−0.054	0.077	0.485
Ego orientation → Physical activity	0.066	0.060	0.269	0.119	0.048	013	0.069	0.107	0.520

All coefficients shown in the table are unstandardized regression coefficients.

## Discussion

The present study extends prior work on adolescent physical activity by elucidating the motivational pathways through which self-efficacy operates. Our findings indicate that self-efficacy directly predicts physical activity and indirectly influences it through both task and ego orientations, with gender moderating the translation of self-efficacy into ego-oriented motives such that this relationship is stronger for boys. Beyond these direct and indirect effects, the two goal orientations appear to operate through distinct socio contextual mechanisms, which we elaborate in the sections that follow.

### Divergent pathways of achievement goals

The direct positive association between self-efficacy and physical activity is consistent with theoretical predictions and prior empirical evidence. Adolescents with stronger efficacy beliefs reported higher levels of physical activity participation. This internal psychological resource enables them to initiate and sustain physical activity despite potential barriers such as time constraints or competing demands.

Our results revealed a strong positive correlation between self-efficacy and task orientation; however, the direct predictive path from task orientation to physical activity did not reach traditional statistical significance in the main structural model. Nevertheless, the bias-corrected bootstrap analysis confirmed a significant specific indirect effect. This statistical nuance can be elucidated by evaluating the overarching life and educational ecology of adolescents. Adolescents holding an incremental belief about ability are intrinsically driven by skill mastery and self-improvement (Vrugt et al., 2002).

The finding that task orientation exerted a significant indirect but non-significant direct effect warrants closer theoretical attention. This pattern suggests that task orientation may function as a conditional mediator: its motivational potential is activated only when accompanied by sufficient self-efficacy. In the absence of strong efficacy beliefs, a mere disposition toward mastery and self-improvement may remain behaviorally inert—particularly within the Chinese educational context, where adolescents’ free time is heavily constrained by academic demands (Zhao et al., 2024). Under such conditions, the intention to improve one’s skills is unlikely to translate into action unless it is coupled with the conviction that one can effectively organize and execute the required behaviors. Thus, self-efficacy serves as the ‘catalyst’ that releases the behavioral potential embedded in task-oriented motivation. This interpretation aligns with Bandura’ (1977) assertion that efficacy beliefs determine whether individuals will translate their intentions into sustained effort, particularly in the face of competing demands.

One plausible explanation for the marginal direct effect of task orientation concerns the broader educational context in which Chinese adolescents are situated. Although task-oriented adolescents possess intrinsic motives for skill mastery and personal improvement, the translation of these motives into regular physical activity may be constrained by competing demands on their time and attention. Prior research has documented that Chinese adolescents face substantial academic pressures and limited opportunities for unstructured leisure time. These contextual factors, although not directly measured in the current study, may attenuate the behavioral expression of mastery-oriented goals. This interpretation remains speculative and requires empirical verification in future research (Steare et al., 2023; Zhao et al., 2024). Beyond time constraints, the pedagogical management climate in Chinese middle schools further complicates this behavioral translation. Daily education predominantly emphasizes discipline, compliance with standardized arrangements, and highly structured, teacher-directed routines (Chen et al., 2020a, 2020b). There is a relative dearth of autonomy-supportive environments that nurture independent exploration and self-determined goal setting in sports (Zhang et al., 2024). Consequently, even when adolescents harbor high task-oriented motives, their reliance on external scheduling may undermine their ability to spontaneously initiate and self-regulate independent physical activity outside of mandatory physical education classes.

In contrast to the marginal direct effect of task orientation, the robust mediating role of ego orientation highlights a context specific motivational adaptation. Self-efficacy significantly predicted ego orientation, which in turn directly and significantly promoted overall physical activity. This pathway appears to reflect the influence of socio cultural factors and the broader peer interaction environment (Gurleyik et al., 2022). In China’s highly performance oriented educational and social climate, adolescents are accustomed to comparing themselves with peers across various domains, including extracurricular activities and social life (Rao, 2008). Within a collectivistic cultural context, ego orientation is often closely tied to group identification and social status (Asghar et al., 2013).

From a developmental perspective, the robust pathway from ego orientation to physical activity may also be understood through the lens of adolescent peer relationships. Early adolescence is a developmental stage characterized by an increased sensitivity to social evaluation and a heightened need for peer acceptance and recognition (Gurleyik et al., 2022). Within the school context, physical competence and athletic performance often serve as visible markers of social status, particularly among male peer groups. For adolescents with high self-efficacy, engaging in physical activity offers an opportunity to demonstrate competence publicly and to gain peer approval, thereby satisfying both achievement-related and social-affiliative needs. In this regard, ego-oriented motivation not only drives behavioral engagement but also aligns with the developmental tasks of early adolescence—namely, establishing social standing and securing peer group membership. This developmental interpretation complements the sociocultural explanation by highlighting the age-specific relevance of competitive motivation during this critical life phase.

For highly efficacious adolescents, physical activity, whether during school recesses or out of school leisure time, serves as a visible platform to demonstrate competence, win peer approval, and establish group standing. Under the current ecology of peer competition and social interaction, ego orientation serves as a salient behavioral driver that encourages adolescents to actively participate in extensive physical activities.

Collectively, these findings suggest that the behavioral translation of achievement goals is contingent upon the surrounding socio ecological context. Task oriented motives may require autonomy supportive environments to manifest in behavior, whereas ego-oriented motives may be more readily activated in competitive peer settings. This differential pattern underscores the importance of considering contextual factors when designing interventions to promote adolescent physical activity.

### Cross-gender invariance in the behavioral translation process

The multi-group structural equation modeling results revealed no statistically significant gender differences in the structural paths from achievement goal orientations to physical activity. That is, although girls reported lower mean levels of self-efficacy, task orientation, and physical activity, the process through which these motivational orientations translate into behavior did not differ between genders. The direct association between self-efficacy and physical activity, as well as the translation pathways from task orientation and ego orientation to physical activity, operated equivalently for boys and girls. This path invariance suggests that once motivational orientations are formed, their behavioral expression does not differ by gender, even though the mean levels of these orientations may differ. From an intervention perspective, this implies that programs aimed at enhancing self-efficacy and fostering adaptive goal orientations may yield comparable behavioral benefits for both boys and girls, provided that the initial mean-level gaps in efficacy and motivation are addressed (Spence et al., 2010). This finding aligns with prior evidence indicating that gender gaps in physical activity participation may be narrowing, at least in terms of motivational processes (Dishman et al., 2013). From an intervention perspective, this invariance implies that programs aimed at enhancing self-efficacy and fostering adaptive goal orientations may yield comparable behavioral benefits for both boys and girls.

It is important to note that the present study adopts a social-cognitive perspective and does not include biological measures such as hormonal levels. While physiological factors (e.g., testosterone) may partially explain gender differences in competitive drive, our findings underscore the sociocultural mechanisms through which gender role expectations shape how adolescents translate efficacy beliefs into performance-oriented motives. Future interdisciplinary research combining psychosocial and biological approaches would be valuable to further elucidate these pathways.

### Gender differences in the formation of Ego orientation

The most prominent gender difference emerged during the motivation elicitation phase. The positive association between self-efficacy and ego orientation was significantly stronger for boys than for their girl counterparts. This finding suggests that when boys experience enhanced efficacy beliefs, they are more likely than girls to translate these beliefs into performance oriented and competitive motives. This pattern may be explained by socio cultural norms and gender role socialization. Cultural expectations often place greater emphasis on competitiveness, strength, and dominance for boys than for girls (Wu et al., 2003). Consequently, boys with high athletic self-efficacy may be more inclined to internalize these normative expectations and express their competence through social comparison and the pursuit of superior performance. For girls, social norms tend to emphasize cooperation and relational harmony, which may channel efficacy beliefs toward less competitive goal orientations. These findings underscore the need for gender sensitive approaches in physical activity interventions that account for the different ways boys and girls construct competence in achievement settings.

### Limitations and future directions

Although this study offers valuable insights, several limitations must be acknowledged. First, the cross-sectional design precludes the determination of causal relationships among the variables. Future longitudinal studies are needed to examine how these structural pathways evolve over time, particularly during developmental transitions such as the move from junior to senior high school.

Second, all variables were assessed using self-report measures, which may introduce recall bias and social desirability effects. Future research could incorporate objective measures, such as accelerometers, to obtain more accurate estimates of adolescents’ physical activity levels.

Third, the International Physical Activity Questionnaire short form, although widely used, was originally developed for adult populations. Its validity among young adolescents, particularly those aged 13 years, has received limited empirical scrutiny. Younger respondents may have difficulty accurately recalling and estimating their physical activity over a seven-day period. Future research should consider using age adapted questionnaires or complementing self-reportdevice-basedevice based measures.

Fourth, we did not collect individual-level socioeconomic status (SES) indicators, such as parental education and household income, which have been shown in some studies to be associated with adolescents’ physical activity participation (Stalsberg and Pedersen, 2010). However, the primary theoretical focus of the present study is on intra-individual psychological processes—specifically, how self-efficacy translates into physical activity through achievement goal orientations—rather than on socio-ecological determinants. Moreover, our sampling strategy, which recruited participants from five cities representing a broad spectrum of economic contexts (with regional GDPs ranging from 21.90 billion to 320.39 billion RMB), partially mitigated the concern regarding SES heterogeneity at the macro level. Nonetheless, future research should incorporate individual-level SES measures to more systematically examine whether and how family background moderates the motivational pathways identified in this study.

Finally, the convenience sampling strategy limits the generalizability of the findings to broader adolescent populations in China. Although the sample size was substantial, participants were recruited from five southern cities, which may not represent adolescents from rural areas or northern regions. Future studies should examine whether the observed mediation patterns, particularly the differential functioning of task and ego orientations, are replicable in countries with different educational systems and cultural backgrounds.

## Conclusion

Self-efficacy promotes adolescent physical activity through direct and dual indirect pathways involving task and ego orientations. The behavioral translation of these achievement goals is dependent on context. Task oriented motives may be constrained by competing academic demands, whereas ego-oriented motives appear to be more readily activated in competitive peer settings. Gender moderates the formation of ego-oriented motives, with boys showing a stronger translation of efficacy beliefs into performance goals, but once activated, these motivational processes operate equivalently across genders. These findings highlight the need for physical activity interventions that address not only individual efficacy beliefs but also the socio contextual conditions that shape adolescents’ achievement goals.

## Data Availability

The datasets presented in this study can be found in online repositories. The names of the repository/repositories and accession number(s) can be found in the article/supplementary material.
